# Early Diagnosis of Pulmonary Tuburculosis

**Published:** 1908-05

**Authors:** 


### EDITOIRALS

The Business Office of the JOURNAL-RECORD is 1 1-2 to 5 1-2 South Broad St.
The Editorial Office is 1014-15 Century Bldg.

Address all Business Communications to Journal-Record of Medicine, 1 1-2 to 5 1-2 South Broad Street.

Make remittances payable to THE JOURNAL-RECORD OF MEDICINE.

On matters pertaining to the Editorial and Original communications, address Edgar G. Ballenger, M. D., Atlanta, Ga.

Reprints of original articles will be furnished at cost price. Requests for the same should always be made in THE MANUSCRIPT.

We will present, postpaid, on request, to each contributor of an original article, twenty (20) marked copies of THE JOUNAL-RECORD OF MEDICINE containing such article.

### EARLY DIAGNOSIS OF PULMONARY TUBERCULOSIS.

The importance of an early diagnosis of pulmonary tuberculosis is so evident to those treating phthisis, and its neglect so common that an excuse for its consideration editorially is not necessary.

When well developed no difficulty whatever is encountered in making a prompt diagnosis. By this time, however, we meet almost insurmountable difficulties in the treatment. The effect of therapeutic measures in its incipiency compared to those when the disease is well developed justifies great stress upon its early recognition.

The demonstration of tubercle bacilli in the sputum is a diagnostic sign of indisputable accuracy, but also means that valuable time has been lost—time in which more sanguine hopes of recovery could have been entertained.

Lawrason Brown* has recently called attention to the question of early diagnosis, and asserts that tuberculosis can often be recognized by the sypmtoms alone, and that the diagnosis must often be so made; for even when the lesion is in the lungs the localizing symptoms may long remain absent. Petruschky distin-

*Albany Medical Annals, April, 1908.

guishes three stages: first, involvement of the bronchial glands; second, involvement of the lung before ulceration; third, the breaking down of the disease focus. Brown insists that pulmonary tuberculosis can exist without producing any physical signs; not that he belittles the importance of physical signs, but urges that we be careful in excluding tuberculosis because no physical signs are present. He has found them characterized, though, chiefly, by their absence, by their occurrence with symptoms, often so slight as to escape our notice, by their slightness even when symptoms have been of long standing, by their variation and evanescence, and by their sudden appearance. They constitute but one of the links in the diagnostic chain.

A careful history and a consideration of the surroundings, failing health and physical signs, if present, often contribute a typical picture.

Rales that can be heard no other way can be made much more apparent by having the patient cough. Occasionally, however, this may cause the rales to disappear.

He thinks that the time will come when the physician who waits for the tubercle bacilli to occur in the sputum will be looked upon as a man deficient in diagnostic acumen and one dangerous for public health. When all known procedures have been carried out and doubt as to the diagnosis still exists the tuberculin test should be suggested. A positive reaction does not prove that the focus is in the lungs, nor does it prove that an apparently healthy individual needs treatment; but when a patient with symptoms suggesting tuberculosis reacts to tuberculin, a course of treatment should be insisted upon.

McClennan* reports one hundred observations with ophthalmo-reaction to tuberculin as introduced last summer by Calmette, of the Pasteur Institute of Lille, as a diagnostic test for the presence of tuberculosis.

It is claimed for this method that it is absolutely safe, easy of application, produces no constitutional disturbance and that it is as accurate as the hypodermic injection if not more so.

The Calmette tuberculin is used in a 1% solution in distilled water. One minim of this is instilled in the inner half of

*British Medical Journal, December 7, 1907.

the conjunctiva. In from three to ten hours, if the reaction be positive, a redness of the conjunctiva is seen. This varies from a slight injection near the caruncle to redness over the entire eye with the symptoms of a conjunctivitis. Of course occular lesions, either acute or chronic contra-indicate its employment.

The analysis of McClennan's cases brings out the facts:

(1) That for the most part the claims advanced by Calmette for his test are fully justified; (2) that the test apparently reveals the presence of tuberculous lesions that are quite benign and unsuspected from a clinical point of view, as well as those that are more obvious; (3) that in those cases in which a subcutaneous injection of "old" tuberculin has given a positive or negative reaction the same result has followed the application of the opthalmic test; (4) there seems some evidence that a solution of the "old" tuberculin may answer equally well.

If this test proves, on further experience, to be reliable, it will be a valuable aid to the early diagnosis in obscure cases. It is now believed that this test is equally as reliable as the hypodermic test, and one worthy of trial.

